# An Overview of Oxidative Stress in Sex Chromosome Aneuploidies in Pediatric Populations

**DOI:** 10.3390/antiox14050531

**Published:** 2025-04-29

**Authors:** Roberto Paparella, Fabiola Panvino, Francesca Tarani, Benedetto D’Agostino, Lucia Leonardi, Giampiero Ferraguti, Sabrina Venditti, Fiorenza Colloridi, Ida Pucarelli, Luigi Tarani, Marco Fiore

**Affiliations:** 1 Department of Maternal Infantile and Urological Sciences, Sapienza University of Rome, 00185 Rome, Italy; roberto.paparella@uniroma1.it (R.P.); francesca.tarani@uniroma1.it (F.T.); benedetto.dagostino@uniroma1.it (B.D.); lucia.leonardi@uniroma1.it (L.L.); fiorenza.colloridi@uniroma1.it (F.C.); ida.pucarelli@uniroma1.it (I.P.); 2 Department of Human Neuroscience, Section of Child and Adolescent Neuropsychiatry, Sapienza University of Rome, 00185 Rome, Italy; fabiola.panvino@uniroma1.it; 3 Department of Experimental Medicine, Sapienza University of Rome, 00185 Rome, Italy; giampiero.ferraguti@uniroma1.it; 4 Department of Biology and Biotechnologies “Charles Darwin”, Sapienza University of Rome, 00185 Rome, Italy; sabrina.venditti@uniroma1.it; 5 Institute of Biochemistry and Cell Biology (IBBC-CNR), Department of Sensory Organs, Sapienza University of Rome, 00185 Rome, Italy

**Keywords:** sex chromosome aneuploidies, antioxidants, oxidative stress, pediatrics, Klinefelter syndrome, Turner syndrome

## Abstract

Background: Oxidative stress, defined as an imbalance between reactive oxygen species and antioxidant defenses, plays a pivotal role in the pathogenesis of sex chromosome aneuploidies (SCAs), such as Turner syndrome (TS) and Klinefelter syndrome (KS). Pediatric patients with SCAs are particularly susceptible due to hormonal deficiencies, metabolic disturbances, and systemic complications. Methods: A comprehensive literature search was conducted in November 2024 using PubMed, Scopus, and Web of Science. Keywords included “antioxidants”, “oxidative stress”, “pediatrics”, “Turner syndrome”, “Klinefelter syndrome”, and “sex chromosome aneuploidies”. English-language articles were included without publication year restrictions. Relevant data on oxidative stress mechanisms and antioxidant interventions were systematically extracted. Results: The relationship between oxidative stress and SCAs can be described as bidirectional, where oxidative stress both contributes to and is exacerbated by aneuploidies. TS is marked by estrogen deficiency, cardiovascular anomalies, and metabolic dysfunction, all linked to heightened oxidative stress. KS is associated with hypogonadism, metabolic syndrome, and neurocognitive challenges, further exacerbated by oxidative damage. The aneuploid condition predisposes to increased oxidative stress in other SCAs, including 47,XXX and 47,XYY, as well as in high-grade aneuploidies. Emerging evidence highlights the therapeutic potential of antioxidants, including vitamin C, vitamin E, glutathione precursors, polyphenols, and melatonin. These interventions, when combined with hormonal therapies such as estrogen replacement in TS or testosterone replacement in KS, demonstrate synergistic effects in restoring redox balance and mitigating systemic complications. Conclusions: Oxidative stress significantly impacts the progression of SCAs in pediatric populations, amplifying risks across metabolic, cardiovascular, and neurocognitive domains. Early, tailored antioxidant strategies, integrated with syndrome-specific hormonal therapies, could reduce long-term complications and improve patient outcomes. Future research should focus on standardizing protocols to optimize these interventions for pediatric patients with SCAs.

## 1. Introduction

Sex chromosome aneuploidies (SCAs) are among the most common chromosomal abnormalities, affecting approximately 1 in 500 live births, with the prevalence varying based on the specific condition [1,2,3]. The most frequent SCAs include Klinefelter syndrome (KS), Turner syndrome (TS), 47,XXX syndrome, and 47,XYY syndrome, along with less common variants and mosaicism forms [4,5].

SCAs are associated with a wide spectrum of clinical manifestations, including developmental delay, cognitive impairment, dysmorphic features, gonadal dysfunction, cardiovascular anomalies, and metabolic disturbances [6,7]. The variability in phenotypic presentation is influenced by the specific aneuploidy, mosaicism degree, and dosage effects of sex chromosome-linked genes [8]. Advances in prenatal diagnostic techniques and improved clinical management have contributed to earlier detection and better outcomes, emphasizing the need for a deeper understanding of underlying pathophysiological mechanisms [9,10,11].

Oxidative stress, characterized by an imbalance between reactive oxygen species (ROS) production and antioxidant defense mechanisms, has been implicated in the pathogenesis of numerous pediatric disorders [12,13]. Emerging evidence suggests that SCAs may also involve oxidative stress, contributing to the development of associated comorbidities such as cardiovascular disease, diabetes, and neurodevelopmental disorders [14,15,16,17]. Notably, oxidative stress has been extensively studied in autosomal aneuploidies such as trisomy 21, where altered redox homeostasis plays a critical role in phenotypic manifestations [18,19]. The role of oxidative stress in SCAs, however, remains underexplored. Specific genes located on sex chromosomes, such as those regulating oxidative balance and mitochondrial function, as well as autosomal genes, may contribute to increased ROS production and decreased antioxidant capacity [20,21,22,23,24,25]. Understanding these mechanisms could illuminate the etiology of SCA-related complications and inform therapeutic strategies targeting oxidative stress.

This review aims to provide an overview of oxidative stress in SCAs in pediatric populations, emphasizing its potential implications for metabolic, cardiovascular, and neurodevelopmental health. Given the limited research specifically addressing oxidative stress in SCAs, we also incorporate relevant findings from autosomal aneuploidies to provide a broader context. Rather than presenting an exhaustive systematic analysis, our goal is to synthesize current knowledge on oxidative stress-related mechanisms in SCAs and discuss potential antioxidant strategies that may contribute to mitigating disease progression. While this review does not claim to exhaustively cover all aspects of SCAs and oxidative stress, it underscores the importance of this research avenue for advancing pediatric and reproductive medicine.

## 2. Materials and Methods

For this narrative review, we conducted a comprehensive literature search using MEDLINE/PubMed, Scopus, and Web of Science to identify studies related to oxidative stress and antioxidant strategies in SCAs, especially in pediatric populations. The search was performed with no restrictions on publication year to ensure the inclusion of all relevant literature. We used the following keywords and Medical Subject Headings (MeSH) terms in various combinations: “Oxidative stress” (unique ID: D015444), “Antioxidants” (unique ID: D000975), “Sex chromosome aneuploidies” (no MeSH term, free text search), “Klinefelter syndrome” (unique ID: D007661), “Turner syndrome” (unique ID: D014424), and “Pediatrics” (unique ID: D010372).

Eligible studies included original research articles (randomized and non-randomized clinical trials, prospective observational studies, retrospective cohort studies, and case–control studies) that investigated oxidative stress and/or antioxidant interventions in SCAs. Additionally, we included review articles discussing oxidative stress mechanisms, antioxidant therapies, or metabolic, cardiovascular, and neurodevelopmental implications in SCAs. Given the limited research on oxidative stress in SCAs, we incorporated case reports and studies on oxidative stress also in autosomal aneuploidies (e.g., trisomy 21) when they provided insights relevant to SCAs. Exclusion criteria were as follows: non-English language manuscripts; articles that did not provide specific data on oxidative stress mechanisms or antioxidant interventions, in particular for pediatric age; and abstract, books, conference proceedings, letters to the editor, and editorials. The selection process was conducted independently by two authors (R.P. and F.Pa.) who screened and reviewed all the studies meeting the inclusion criteria. A total of 161 articles were included, covering oxidative stress mechanisms, antioxidant strategies, and potential clinical implications in pediatric SCAs.

## 3. Overview of ROS

ROS refer to oxygen-containing molecules that are more reactive than molecular oxygen (O_2_) [26]. They arise due to the partial reduction of oxygen during metabolic processes, such as mitochondrial respiration [27]. The term encompasses both free radicals (molecules with one or more unpaired electrons) and non-radical derivatives of oxygen that exhibit high reactivity [28]. Key examples of ROS include the following:

- Superoxide anion (O_2_^•−^): a free radical formed by the one-electron reduction of oxygen [29].
- Hydrogen peroxide (H_2_O_2_): a non-radical ROS that can diffuse through membranes and act as a signaling molecule [30].
- Hydroxyl radical (^•^OH): an extremely reactive free radical generated from H_2_O_2_ via the Fenton reaction [31].
- Singlet oxygen (^1^O_2_): a highly reactive non-radical form of oxygen produced by energy transfer to molecular oxygen [32].
- Peroxynitrite (ONOO^−^): formed by the reaction of nitric oxide (NO) with superoxide; it is classified as a reactive nitrogen species but often grouped with ROS [33].

**Figure 1:**
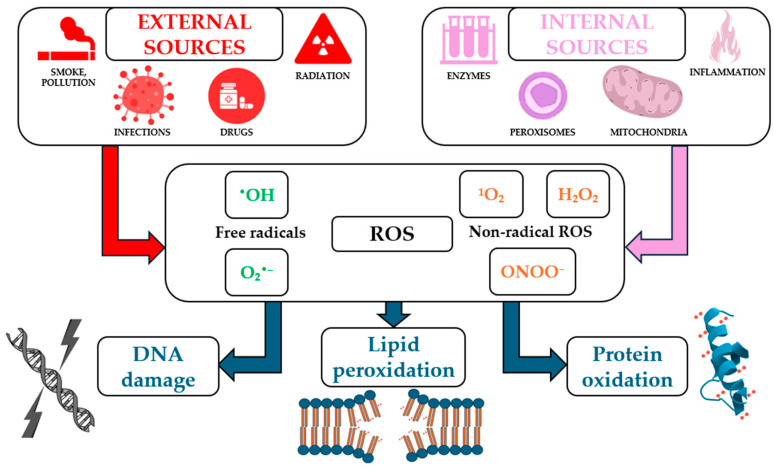
Main internal and external sources of reactive oxygen species (ROS) and their biological effects (not all the mechanisms illustrated in the figure have been detailed or discussed in the text of the article).

ROS can arise from internal sources such as mitochondrial electron transport, peroxisomal oxidases, and nicotinamide adenine dinucleotide phosphate (NADPH) oxidases (NOX), as well as from external sources, including ultraviolet (UV) radiation, pollution, and certain drugs (Figure 1). They serve dual roles in biological systems, acting as critical signaling molecules under physiological conditions and as damaging agents under pathological circumstances [34].

In controlled amounts, ROS are essential for various cellular processes:

- Signal transduction: ROS, particularly H_2_O_2_, function as secondary messengers in signaling pathways [35]. For instance, H_2_O_2_ can reversibly oxidize thiol groups in proteins, regulating kinases and phosphatases involved in growth, differentiation, and immune responses [36].
- Host defense: ROS generated by phagocytes (via NOX enzymes) are crucial for destroying pathogens. This process, known as the respiratory burst, produces O_2_^•−^, which is converted into bactericidal species such as hypochlorous acid (HOCl) [37].
- Redox homeostasis: they regulate the expression of antioxidant genes through redox-sensitive transcription factors like nuclear factor erythroid 2-related factor 2 [38].
- Cellular adaptation to stress: moderate ROS levels activate stress response pathways, such as heat shock proteins and autophagy, promoting cell survival [39].

Excessive ROS production or impaired antioxidant defenses lead to oxidative stress, contributing to numerous diseases, including neurodegenerative disorders, cancer, cardiovascular diseases, and chronic inflammation [34]. Moreover, the free radical theory of aging suggests that accumulated oxidative damage over time contributes to cellular senescence and organismal aging [40].

The chemical reactions involving ROS are diverse and depend on the type of ROS and their specific biological or chemical context. The key reactions can be categorized as generation, conversion, and interaction with biological molecules.

### 3.1. Generation Reactions

ROS are produced through various physiological and environmental processes, in both enzymatic and non-enzymatic reactions.

Enzymatic production

- NOX: these enzymes catalyze the transfer of electrons from NADPH to molecular oxygen, forming O_2_^•−^ [41].
- Xanthine oxidase: produces O_2_^•−^ and H_2_O_2_ during purine metabolism [42].
- Cytochrome P450 enzymes: generate O_2_^•−^ as a byproduct during detoxification reactions [43].

Non-enzymatic production

- Electron leakage from the mitochondrial electron transport chain during oxidative phosphorylation can reduce oxygen to O_2_^•−^ [44].
- UV radiation and ionizing radiation split water molecules, producing ^•^OH [45,46].

### 3.2. Conversion Reactions

Once generated, ROS are often converted into other species via chemical or enzymatic reactions:

- Superoxide dismutase (SOD) converts O_2_^•−^ into H_2_O_2_: 2O_2_^•−^ + 2H^+^ → H_2_O_2_ + O_2_ [47].
- Fenton reaction: H_2_O_2_ reacts with transition metals (e.g., Fe^2+^), producing ^•^OH: H_2_O_2_ + Fe^2+^ → ^•^OH + OH^−^ + Fe^3+^ [48].
- Haber-Weiss reaction: the reaction between O_2_^•−^ and H_2_O_2_ to produce ^•^OH, which occurs in the presence of metal ions, particularly iron (Fe^2+^ or Fe^3+^): O_2_^•−^ + H_2_O_2_ → ^•^OH + OH^−^ + O_2_ [49].

### 3.3. Interactions with Biological Molecules

ROS react with lipids, proteins, and DNA, leading to significant biological effects [50]:

- Lipid peroxidation: lipid radicals and malondialdehyde (MDA) are generated, disrupting membrane integrity [51].
- Protein oxidation: oxidation of amino acids, such as cysteine and methionine, can modify enzyme activity and signaling pathways [52].
- DNA damage: ROS can induce strand breaks and base modifications, such as the conversion of guanine to 8-oxoguanine, contributing to mutagenesis [53].

## 4. Parental Meiotic Errors and Offspring Susceptibility to Oxidative Stress in SCAs

SCAs arise from meiotic nondisjunction, which can occur in both maternal and paternal meiosis, although maternal nondisjunction is more common. Errors in the first and second meiotic divisions, influenced by factors such as recombination failures and maternal age, are primary contributors to these aneuploidies. KS (47,XXY) can arise from nondisjunction in either parent, with approximately equal contributions from maternal or paternal origins [54,55]. 47,XXX syndrome predominantly results from maternal nondisjunction, with a small percentage arising from paternal errors [54,55]. 47,XYY syndrome usually results from nondisjunction during paternal meiosis II [55,56]. TS, in contrast, often results from postzygotic mitotic errors or nondisjunction during paternal meiosis, leading to different karyotypes, all of which lack X-chromosomal material [57]. More complex SCAs include the high-grade sex chromosome aneuploidies (HGAs) with more than 47 chromosomes that arise from a maternal or paternal non-disjunction event during meiosis I or II [58]. The distinct mechanisms underlying these errors include improper chromosomal pairing, cohesion defects, and spindle assembly checkpoint failures [59]. These disruptions lead to aneuploid gametes, which, when involved in fertilization, result in offspring with SCAs.

Once SCAs are established in the zygote, the aneuploid condition, as well as the number of X chromosomes, may predispose the offspring to increased oxidative stress (Table 1). It is hypothesized that the chromosomal imbalance alters gene expression profiles, particularly in genes involved in redox regulation, further increasing the susceptibility of cells to oxidative damage. SCAs impact cellular and tissue function through both cis and trans gene expression effects [60]. Cis effects, particularly on X-Y gametologs (homologous genes on X and Y chromosomes that evolved from a single ancestral gene and retained related functions [61]), are consistent across tissue types, influencing critical processes like gene regulation and chromatin dynamics. In contrast, trans effects exhibit tissue specificity, potentially driving varied clinical outcomes in neurodevelopment, reproduction, and metabolism [60]. Therefore, the potential oxidative imbalance likely exacerbates the various comorbidities associated with SCAs [62].

While parental meiotic errors are the primary cause of SCAs, the resultant chromosomal abnormalities set the stage for oxidative stress-mediated pathogenesis in the offspring. The supernumerary sex chromosomes carry imprinted genes that influence cellular metabolism and ROS production, linking the meiotic origin of SCAs with the downstream oxidative stress observed in affected individuals.

The degree of oxidative stress susceptibility appears to vary depending on the specific aneuploidy and the genes involved [60]. For example, the presence of an extra X chromosome in 47,XXY, or 47,XXX may increase the gene dosage of X-linked pro-oxidant genes that escape X-inactivation. In 47,XYY individuals, for instance, the Y-linked gene involvement in redox regulation may have a role. However, the evidence is not uniformly consistent. Thus, while gene dosage and meiotic origin clearly contribute to oxidative vulnerability, the precise degree and mechanisms remain incompletely defined and are likely modulated by epigenetic, environmental, and sex-specific factors.

**Table 1:** Main oxidative stress findings across sex chromosome aneuploidies.

	Karyotypes	Key Findings on Oxidative Stress	References
Single X Chromosome	45,X (Turner syndrome, TS)	Reduced antioxidant capacity, increased oxidative stress markers (e.g., lipid peroxidation, reduced glutathione levels)—see Table 2	[63,64]
	Mosaic TS (e.g., 45,X/46,XX)	Oxidative stress may vary depending on the degree of mosaicism; similar trends to 45,X are observed	
	Mosaic TS with Y material (e.g., 45,X/46,XY)	Similar to 45,X, but limited data on specific oxidative stress markers in this subgroup	
	47,XYY	Limited evidence: oxidative stress not well characterized in this aneuploidy	[65], “Limited data”
Multiple X Chromosomes	47,XXY (Klinefelter syndrome)	Increased levels of oxidative stress biomarkers; associated with metabolic syndrome, which exacerbates oxidative stress—see Table 2	[65,66,67]
	47,XXX	Limited studies: oxidative imbalance likely less pronounced than in other sex chromosome aneuploidies	[68], “Limited data”
	Tetrasomies and pentasomies with supernumerary X (and/or Y) chromosomes	Insufficient data; presumed oxidative stress due to multiple X (and/or Y) chromosomes and severe aneuploidy effects	[69], “Limited data”

**Table 2:** Oxidative stress mechanisms and impact in Turner syndrome and Klinefelter syndrome. Abbreviations: ROS, reactive oxygen species; XIAP, X-chromosome-linked inhibitor of apoptosis protein; SOD, superoxide dismutase; NADPH, nicotinamide adenine dinucleotide phosphate; NOX, NADPH oxidase; GPx, glutathione peroxidase.

	Turner Syndrome	Klinefelter Syndrome
Genetic Alteration	Lack of X chromosome material (45,X or different karyotypes) increases susceptibility to oxidative stress.	Extra X chromosomes (most common karyotype: 47,XXY) lead to increased ROS production and reduced antioxidant capacity.
Oxidative Stress Response	Altered stress pathways, with increased oxidative burden due to estrogen deficiency.	Elevated ROS and impaired antioxidant defense, especially in spermatozoa.
Molecular Mechanisms	XIAP regulates mitochondrial antioxidants (SOD-2), reducing oxidative stress.	Increased NADPH production enhances ROS generation via sperm NOX.
Antioxidant Defenses	Estrogen upregulates SOD, catalase, and scavenges free radicals; estrogen replacement may help restore defenses.	Testosterone modulates antioxidant enzymes (SOD, GPx), with testosterone replacement improving oxidative stress.
Cardiovascular Implications	Oxidative stress leads to endothelial dysfunction, hypertension, and aortic issues.	ROS in endothelial cells cause dysfunction, contributing to cardiovascular risk.
Metabolic Issues	Oxidative stress impairs insulin signaling and promotes metabolic syndrome.	Excess adipose ROS exacerbates metabolic dysfunction and systemic inflammation.
Neurodevelopmental Challenges	Oxidative stress in the brain may contribute to cognitive and developmental deficits.	Mitochondrial dysfunction and ROS accumulation affect cognitive development.

## 5. SCAs and Oxidative Stress in Pediatric Populations

Oxidative stress results from an imbalance between the production of ROS and the body’s ability to neutralize them through antioxidants. Excessive ROS can damage lipids, proteins, and DNA, leading to cellular dysfunction and contributing to the development of chronic diseases [50]. Recent studies have suggested that the altered gene dosage associated with SCAs may result in disrupted metabolic and oxidative processes, offering insight into the pathogenic mechanisms of these syndromes [66,67].

Research indicates that oxidative stress markers differ between sexes, with boys exhibiting higher levels of oxidative stress markers and lower levels of antioxidant defenses compared to girls during the neonatal period. This discrepancy is attributed to the more active estrogen metabolism in females, which enhances glutathione metabolism, a critical antioxidant pathway [70].

These findings suggest that antioxidant strategies should be personalized, considering the sex of the individual to effectively restore redox homeostasis. Investigating oxidative stress markers in SCAs has been proposed as a promising avenue to elucidate these processes.

### 5.1. Turner Syndrome

TS is a genetic condition caused by the complete or partial absence of one X chromosome, which affects approximately 1 in 2500 live female births [71]. It encompasses a range of karyotypes, with 40–50% of cases presenting as 45,X (complete loss of one X chromosome). Mosaic forms, such as 45,X/46,XX or 45,X/47,XXX, occur in 15–25% of cases. Isochromosomes are found in 20% of cases, and ring chromosomes are relatively rare. Additionally, 10–12% of individuals possess varying amounts of Y chromosome material, with about 3% showing a 45,X/46,XY mosaic karyotype [57]. While its clinical presentation is diverse—ranging from short stature and ovarian insufficiency to cardiovascular anomalies and metabolic dysfunction [57]—there is growing evidence that oxidative stress plays a central role in the pathogenesis and progression of many complications associated with TS [63] (Table 2). This underscores the importance of understanding oxidative stress mechanisms and exploring antioxidant-based strategies, particularly in pediatric and adolescent populations.

TS exhibits a distinct oxidative stress response compared to normal 46,XX cells. Transcriptomic analysis reveals that over 350 transcripts are differentially expressed in 45,X cells under mild oxidative stress conditions. These cells show increased susceptibility to oxidative stress and altered regulation of stress-related molecular pathways. The differential expression of transcription factors in 45,X cells suggests that the altered stress response may contribute to the various phenotypes and comorbidities observed in TS, such as short stature, osteoporosis, and ovarian malfunction [63]. Furthermore, the X-chromosome plays a significant role in managing oxidative stress through mechanisms involving the X-chromosome-linked inhibitor of apoptosis protein (XIAP), encoded by the *XIAP* gene, and its regulation of mitochondrial antioxidants. XIAP has been shown to play a crucial role in reducing oxidative stress in brain injury models, such as hypoxia–ischemia and cerebral irradiation. Its overexpression leads to a significant decrease in oxidative stress markers by upregulating certain mitochondrial antioxidants, including SOD-2. This upregulation helps in reducing cytochrome c release from mitochondria, thereby mitigating oxidative damage [72]. An additional factor potentially influencing oxidative stress in TS is the embryonic origin of the condition. TS cases derived from 46,XY embryos may differ in their oxidative stress response compared to those derived from 46,XX embryos, given differences in Y-linked gene expression during early development. However, current evidence addressing this distinction is scarce, and further research is needed to determine whether embryonic origin impacts oxidative stress susceptibility in TS.

Estrogen deficiency, a hallmark of TS due to ovarian dysgenesis, has been implicated in increased oxidative stress [73]. Estrogens exert antioxidant effects by upregulating the expression of antioxidant enzymes like SOD and catalase, as well as by directly scavenging free radicals [74]. The absence of these protective effects in TS may lead to a higher oxidative burden, particularly during adolescence, a critical period for hormonal regulation. Based on the results of studies on estrogen replacement therapy (ERT) in postmenopausal women [75], it is plausible to state that it could partially restore antioxidant defenses, particularly when initiated early in adolescence; however, its role in oxidative stress management warrants further investigation [76].

The synergy between ERT and antioxidant therapies likely involves estrogen-mediated activation of redox-sensitive transcription factors (e.g., Nrf2), which upregulate SOD, glutathione peroxidase (GPx), and catalase [77]. Estrogens also stabilize mitochondrial membranes, reducing ROS leakage [78]. ERT may thus potentiate the effects of dietary antioxidants by enhancing endogenous antioxidant gene expression [79].

Girls with TS are predisposed to congenital heart defects (e.g., bicuspid aortic valve, coarctation of the aorta) and acquired cardiovascular complications, such as hypertension and aortic dilation or dissection [71]. These conditions are closely associated with oxidative stress, which promotes endothelial dysfunction, inflammation, and vascular remodeling. Elevated ROS in vascular tissues induce endothelial dysfunction by impairing NO bioavailability and promoting inflammation, thrombosis, and arterial stiffness [80]. Soto and colleagues demonstrated significant alterations in antioxidant enzyme activities, particularly in SOD isoforms, in the aortic tissue of patients with TS, suggesting a compensatory response to oxidative stress. Moreover, an impaired antioxidant defense system was depicted in the presence of heightened oxidative damage with increased levels of lipid peroxidation markers such as MDA. A decreased expression of endothelial NO synthase, which could contribute to endothelial dysfunction and aortic complications, was also found [64].

Patients with TS are at increased risk for metabolic syndrome, including insulin resistance, dyslipidemia, and central adiposity, even in pediatric age [81]. Oxidative stress has a well-established role in impairing insulin signaling pathways and contributing to β-cell dysfunction [82]. Moreover, adipose tissue is a significant source of ROS, perpetuating oxidative damage in metabolic pathways and contributing to chronic low-grade inflammation [83]. This is particularly concerning during adolescence, as the onset of metabolic complications often coincides with puberty. The interplay between oxidative stress and chronic low-grade inflammation in TS exacerbates metabolic risks, making early intervention essential.

Cognitive challenges and neurodevelopmental differences, particularly in visuospatial processing and executive function, are frequent in TS [71]. Emerging research suggests that oxidative stress in the brain, through mitochondrial dysfunction and neuroinflammation, in the form of ROS accumulation in neural tissues, may contribute to these deficits [84]. Pediatric populations may be particularly vulnerable, as the developing brain is highly sensitive to oxidative damage [85,86,87].

### 5.2. Klinefelter Syndrome

KS is characterized by the presence of one or more extra X chromosomes in males (47,XXY being the most common karyotype) and affects approximately 1 in 500–660 live male births [88]. While KS is often recognized for its impact on fertility and hypogonadism, it also involves a broad range of systemic complications, including metabolic syndrome, cardiovascular diseases, and neurodevelopmental challenges, with numerous biomarkers potentially predictive or prognostic of alterations strictly connected to the syndrome [89,90,91,92]. Oxidative stress has emerged as a key contributor to these complications (Table 2). The additional genetic material can lead to increased ROS production and reduced antioxidant capacity, contributing to defining the typical phenotype of KS [66]. Additionally, oxidative stress is linked to male infertility through the generation of excess free radicals by spermatozoa, which may result from defective Sertoli cell function, a characteristic shared with KS. This defect leads to inadequate removal of residual cytoplasm during sperm maturation, leaving excess cytoplasmic components that enhance free radical production. One potential mechanism involves elevated glucose-6-phosphate dehydrogenase levels, which increase NADPH production, fueling free radical generation via sperm NOX [65]. However, further research is needed to clarify the exact mechanisms involved.

The oxidative stress in patients with KS can further complicate their clinical management and overall health outcomes [80]. Understanding its role in KS, particularly during the pediatric and adolescent years, is critical for developing targeted antioxidant strategies that may mitigate long-term health risks [93].

Testosterone plays an important role in maintaining redox balance through its influence on antioxidant enzyme activity, with both pro-oxidant and antioxidant effects [94]. Research indicates that testosterone modulates the endogenous antioxidant systems, impacting the activity and expression of cellular antioxidants. For instance, testosterone signaling via the androgen receptor has been shown to have both pro- and antioxidant effects on the heart, influencing the natural antioxidant system glutathione [95]. Furthermore, androgen deprivation in the rat prostate has been shown to induce oxidative stress through the elevation of ROS and the reduction of key ROS-detoxifying enzymes such as SOD and GPx. Testosterone replacement therapy (TRT) in these cases partially restored the expression of these antioxidant enzymes, indicating its potential therapeutic role in reducing oxidative stress [96]. In cases of male secondary hypogonadism, TRT has been observed to significantly enhance levels of coenzyme Q10 and total antioxidant capacity, suggesting an interrelationship between different antioxidants and a potential reduction in oxidative stress [97]. Additionally, the management of oxidative stress in male hypogonadism, particularly in the context of age-related non-communicable chronic diseases, may benefit from conventional hormone replacement therapy, dietary antioxidant supplementation, and lifestyle changes [98]. In summary, testosterone plays a significant role in regulating oxidative stress and antioxidant defenses. Hypogonadism, as seen in KS, leads to reduced antioxidant capacity. The hypogonadism observed in KS leads to reduced antioxidant defenses, such as decreased levels of SOD and GPx, contributing to heightened oxidative stress, but TRT may offer a partial restoration of these defenses, highlighting its potential therapeutic benefits. Testosterone replacement appears to exert its antioxidant effects by also modulating NOX activity and enhancing the expression of mitochondrial antioxidant enzymes. This hormonal–oxidative stress interaction may restore redox homeostasis in KS, particularly in tissues such as testis, adipose, and neural tissue [96]. Co-administration of antioxidants with TRT may optimize therapeutic outcomes.

KS is strongly associated with obesity and dyslipidemia, which are linked to oxidative stress. As abovementioned, excess adipose tissue generates ROS, which exacerbates systemic inflammation and metabolic dysfunction [83]. Pediatric patients with KS often display early signs of metabolic syndrome, highlighting the need for timely interventions [99]. Endothelial dysfunction and insulin resistance are also common in KS, even in very young subjects, and contribute to the elevated risk of cardiovascular disease [100]. Oxidative damage to endothelial cells promotes inflammation, thrombosis, and vascular remodeling, amplifying cardiovascular risks even in children and adolescents [80].

Neurodevelopmental challenges, including deficits in executive function, language processing, and social cognition, are frequently seen in pediatric patients with KS [90,101,102]. Mitochondrial dysfunction and oxidative stress in neural tissues may underlie these deficits, particularly during critical periods of brain development in childhood and adolescence. The aforementioned neurological alterations resulting from oxidative stress in TS also apply to KS [84,85,86,87].

### 5.3. 47,XXX, 47,XYY, and HGAs

47,XXX (or triple X or trisomy X) syndrome affects approximately 1 in 1000 female births. While many individuals with this condition are asymptomatic, children with 47,XXX syndrome commonly present with language and motor delays, hypotonia, cognitive deficits, learning disabilities, and psychological disorders including depression, anxiety, and attention deficits [68]. 47,XYY (or Jacob) syndrome, which occurs in approximately 1 in 1000 male births, is also often underdiagnosed [103]. Common clinical features include tall stature, learning disabilities, mild developmental delays, and behavioral problems, including a higher prevalence of attention-deficit/hyperactivity disorder and autism spectrum disorders [104]. Overexpression of the genes escaping X inactivation might account for the phenotypic differences observed in 47,XXX syndrome, just as the 47,XYY phenotype could primarily be attributed to the abnormal gene dosage resulting from the extra Y chromosome [105]. Tetrasomies and pentasomies with supernumerary X and/or Y chromosomes are extremely rare and tend to present with more severe clinical features, including intellectual disability, growth abnormalities, and significant neuropsychiatric disturbances [55,69,106,107].

While clinical descriptions of 47,XXX and 47,XYY syndromes are relatively well established, evidence regarding oxidative stress in these and HGAs remains sparse and largely speculative. Due to the rarity of these conditions and the paucity of dedicated studies, the following considerations are primarily hypothetical and should be interpreted with caution.

Though oxidative stress has not represented a primary focus in studies of HGAs, 47,XXX, and 47,XYY syndromes, emerging research indicates that the relationship between oxidative stress and SCAs can be described as bidirectional, where oxidative stress both contributes to and is exacerbated by aneuploidies Some proposed mechanisms, based primarily on experimental and in vitro models, suggest that oxidative stress induces ploidy changes through disruption of spindle assembly checkpoint function, energy shortages, centrosome over-replication, and microtubule-kinetochore disorganization [108].

A recent hypothesis suggests that oxidative modifications of proteins crucial for mitosis and the cytoskeleton might lead to disruptions in the cell cycle and interfere with cell division [109]. ROS have been shown to alter structural proteins such as vimentin, actin, and tubulin, which can disrupt the formation of the spindle apparatus, cause chromosome misalignment, and result in failure of cytokinesis [110,111]. On the flip side, the presence of aneuploidy, including SCAs, creates cellular imbalances that might contribute to further oxidative stress. Cells with abnormal chromosome numbers may experience increased metabolic stress and mitochondrial dysfunction; for example, the extra genetic material in individuals with trisomies (or tetrasomies or pentasomies) may lead to cellular stress, increasing the production of ROS and further aggravating oxidative damage [112]. This may suggest a potentially vicious cycle in which oxidative stress contributes to chromosomal instability, and chromosomal abnormalities in turn promote oxidative damage. This reciprocal relationship suggests that both oxidative stress and SCAs can influence each other, potentially amplifying the effects of each and contributing to the cellular dysfunction observed in these conditions.

While these mechanisms are biologically plausible, the limited number of dedicated studies and the small sample sizes available prevent definitive conclusions. Phenotypic variability, mosaicism, and environmental modifiers further complicate interpretation. Given the speculative nature of current hypotheses, further research using well-powered, phenotype-stratified studies is essential to determine whether oxidative stress plays a causative or merely correlative role in HGAs.

### 5.4. Sex Chromosome-Linked and Autosomal Gene Contributors to Oxidative Stress in SCAs

Understanding the genetic contributors to oxidative stress in SCAs is crucial for developing targeted therapeutic strategies. Among the mechanisms discussed, X-linked genes, such as *XIAP*, are key players in modulating oxidative stress. *XIAP* plays an essential role in inhibiting apoptosis by directly binding to and inhibiting caspases, thereby protecting cells from oxidative damage. Reduced expression of *XIAP* has been associated with increased vulnerability to oxidative stress in TS, as the protective effect of this gene is diminished due to the lack of a second X chromosome [22,72]. In addition to caspase inhibition, *XIAP* also promotes the expression of antioxidant enzymes such as SOD-2, with its dysfunction being particularly relevant in pediatric tissues, especially the brain and cardiovascular system [63] (Table 3).

In addition to *XIAP*, several other X-linked genes are implicated in oxidative stress regulation. *SLC25A5*, which encodes a mitochondrial adenine nucleotide translocator, is critical for ATP/ADP exchange across the mitochondrial membrane. Dysregulation of *SLC25A5* leads to an imbalance in mitochondrial function and excessive production of ROS, potentially contributing to oxidative stress in SCAs [23,113]. This pattern has been confirmed in fibroblast models from Turner syndrome patients [63]. Moreover, mitochondrial dysfunction is frequently reported in SCAs [20], and the interplay between mitochondrial and sex chromosome-linked genetic factors remains poorly understood. Emerging evidence suggests that sex chromosome-linked genes, such as *SLC25A5*, may mediate mitochondrial dysfunction in SCAs [23,113].

Transcription factors encoded by *EGR1*, *KLF4*, and *SOX11* are also differentially expressed in X monosomy and participate in multiple oxidative stress-related pathways. *EGR1* is associated with cell proliferation and apoptosis regulation and acts as a central hub in Turner-associated gene networks. *KLF4* and *KLF2*, which encode two mechanosensitive transcription factors, regulate endothelial homeostasis and antioxidant responses; their downregulation may contribute to the increased vascular risk observed in SCAs. Meanwhile, *SOX11* is involved in osteogenic and neurodevelopmental processes and is overexpressed in TS, where it may underlie characteristic skeletal anomalies [63].

The interplay among the Y chromosome, its genes, and oxidative stress is complex and multifaceted. While significant progress has been made in understanding their roles in male infertility and other health issues, further research is essential to unravel the underlying mechanisms and develop effective interventions. The Y chromosome, through genes such as sex-determining region Y (*SRY*), regulates the expression of X-linked genes like monoamine oxidase A (*MAO-A*), which plays a key role in brain function and development. This *SRY*-mediated regulation of *MAO A* highlights the broader influence of the Y chromosome beyond its role in reproduction, potentially affecting cellular responses to oxidative stress and contributing to sex-specific differences in redox homeostasis [24]. MAO-A generates H_2_O_2_ during the catabolism of catecholamines [114], and chronic accumulation of ROS may contribute to neurodevelopmental disorders such as attention-deficit/hyperactivity disorder and autism spectrum disorders in 47,XYY and 47,XXY individuals.

Notably, oxidative stress in SCAs is not solely influenced by genes on the sex chromosomes. Mitochondrial function plays a pivotal role in ROS production, and mitochondrial dysfunction is frequently reported in SCAs [20]. Genes not located on the sex chromosomes also contribute significantly to oxidative homeostasis. One such gene is *GPX4*, which encodes GPx-4, an antioxidant enzyme that prevents ferroptosis by reducing phospholipid peroxides. Although not SCA-specific, its activity is crucial in protecting tissues such as the brain and testes, and its dysfunction may be particularly relevant in KS, where oxidative stress may exacerbate testicular and cognitive impairment [115].

Moreover, the upregulation of *NOX4*, a member of the NADPH oxidase family responsible for H_2_O_2_ production, has been documented in both 45,X and 46,XX cells under oxidative stress, suggesting a role in metabolic dysregulation and thyroid dysfunction [63]. Other autosomal stress-responsive genes of interest include *GADD45B*, which modulates mitogen-activated protein kinase (MAPK) signaling and is implicated in inflammation and cancer progression, and *DUOXA1*, a maturation factor for dual oxidase (DUOX) enzymes involved in thyroid ROS metabolism. Downregulation of *DUOXA1* may contribute to the thyroid abnormalities frequently reported in Turner syndrome [63].

Furthermore, oxidative stress has been identified as a potential underlying cause of idiopathic premature ovarian failure (POF). Women with POF—where X-chromosome anomalies play a significant role in the pathogenesis—exhibit significantly higher levels of ROS compared to controls, suggesting that oxidative stress may contribute to ovarian dysfunction [25]. It is also worth noting that even a slight increase in ROS levels during early development may elevate the risk of SCAs. In mouse models, increased oxidative stress during the first mitotic divisions has been shown to promote chromosomal missegregation, highlighting the importance of centromeric proteins and genomic stability in SCA pathogenesis [21].

**Figure 2:**
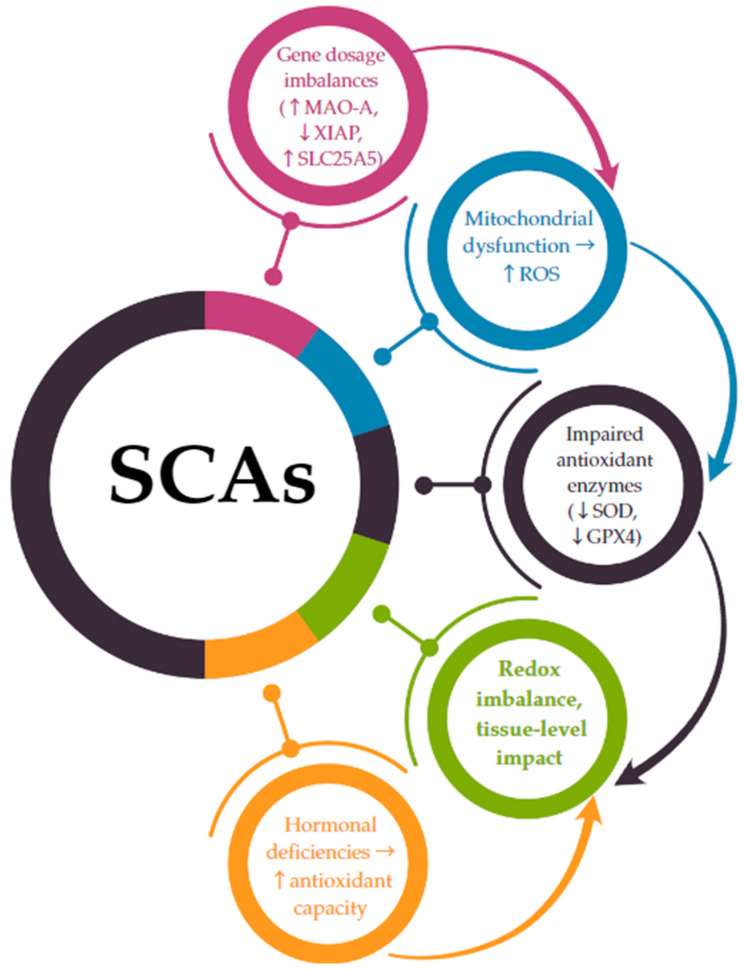
A mechanistic link between SCA-related gene dosage and oxidative stress imbalance. ↑ = increase; ↓ = decrease; → = leads to.

The connection between chromosomal aneuploidy and oxidative stress is increasingly supported by transcriptomic and functional studies showing that gene dosage alterations directly impact redox-sensitive pathways [116]. In SCAs, this relationship is not merely associative but may be causal, as several X- and Y-linked genes implicated in oxidative homeostasis escape X-inactivation or are overexpressed due to chromosomal gain [22,23,24]. A hypothetical model integrating genetic, mitochondrial, and hormonal factors suggests a direct mechanistic link between SCAs and oxidative stress imbalance. The following pathway is proposed (Figure 2):

- SCAs lead to gene dosage imbalances due to monosomy (e.g., 45,X) or the presence of supernumerary chromosomes (e.g., 47,XXY; 47,XYY).
- Genes that escape X-inactivation or are overexpressed on the Y chromosome (such as *MAO-A*, *SLC25A5*, and *XIAP*) are dysregulated, disrupting mitochondrial function and increasing ROS production.
- These changes are compounded by a reduced antioxidant enzymatic defense (e.g., decreased expression of SOD, GPX4).
- The resulting redox imbalance contributes to cellular and systemic dysfunction, particularly in cardiovascular, metabolic, and neurodevelopmental systems.

Hormonal deficiencies, notably estrogen in TS and testosterone in KS, exacerbate the impaired redox balance by failing to adequately support antioxidant pathways.

While some aspects of this model remain hypothetical, the accumulation of gene expression, molecular, and clinical data offers a compelling basis for future translational research. Future research should aim to clarify the contributions of these sex chromosome-linked and autosomal genes to oxidative stress and explore potential therapeutic interventions. Antioxidant therapies, such as N-acetylcysteine and vitamin E, may provide a means to mitigate oxidative damage in individuals with SCAs. Additionally, personalized approaches targeting specific genetic vulnerabilities, such as *XIAP* or *SLC25A5*, could improve outcomes in these patients.

### 5.5. Comparative Insights: Oxidative Stress in Other Pediatric Genetic Syndromes

While oxidative stress appears to be a hallmark of SCAs due to gene dosage imbalance and hormonal deficiencies, it is also a common pathogenic feature in various autosomal genetic syndromes, often contributing to the clinical phenotype through mitochondrial dysfunction, inflammation, and impaired antioxidant responses.

A well-characterized example is Down syndrome, in which the triplication of chromosome 21 leads to overexpression of genes involved in redox regulation. Notably, the *SOD1* gene, encoding superoxide dismutase 1, is located on chromosome 21 and is overexpressed in these individuals. This results in an increased dismutation of O_2_^•−^ into H_2_O_2_, which, if not adequately neutralized by catalase or GPx, leads to the accumulation of ROS and oxidative damage to cellular components such as DNA, proteins, and lipids [18,19]. Mitochondrial dysfunction is also a major contributor to oxidative stress in Down syndrome and has been implicated in the early onset of Alzheimer’s disease and neurodegeneration observed in this population [18].

Another illustrative condition is Williams–Beuren syndrome, caused by a microdeletion at 7q11.23 and characterized by cardiovascular abnormalities, connective tissue disorders, and cognitive deficits. Recent evidence suggests that elastin haploinsufficiency contributes to vascular oxidative stress through increased NOX activity and reduced endothelial NO availability, resulting in endothelial dysfunction and inflammation [117,118]. Elevated markers of lipid peroxidation and reduced glutathione levels have been reported in both serum and vascular tissues of these patients, highlighting a systemic oxidative imbalance [119].

These comparisons underscore that oxidative stress is not exclusive to SCAs but rather a shared pathogenic mechanism across various genetic syndromes. However, the molecular underpinnings differ considerably. In Down syndrome, the imbalance stems from excess antioxidant enzyme activity and mitochondrial vulnerability, while in Williams–Beuren syndrome, the loss of vascular structural integrity and redox regulation is central. In SCAs, oxidative stress arises from altered expression of sex chromosome-linked genes and hormonal dysregulation. Understanding these differences is essential for developing syndrome-specific antioxidant strategies.

## 6. Supplementing Dietary Antioxidants as a Therapeutic Strategy

While the underlying mechanisms and clinical presentations differ between syndromes, antioxidant dietary strategies can provide a common therapeutic approach to mitigate oxidative damage and improve health outcomes in pediatric and adolescent patients [120]. However, it is important to recognize that nutritional needs, antioxidant metabolism, and optimal dosages may vary by age and sex (and karyotype) (Table 4). To enhance clinical applicability, dietary reference values (DRVs) for pediatric populations are included wherever available, particularly highlighting differences between males and females. While precise recommendations tailored to each specific SCA remain under-researched, these general guidelines can provide a foundation for appropriate supplementation in clinical practice.

Vitamin C, a potent water-soluble antioxidant, neutralizes ROS and regenerates other antioxidants like vitamin E [121]. It supports vascular health by enhancing endothelial function and reducing inflammation [122], which is crucial for both TS, where cardiovascular anomalies such as aortic coarctation are prevalent [123], and KS, which is associated with endothelial dysfunction and increased cardiovascular risks [124]. Supplementation with vitamin C may also help address metabolic complications, including insulin resistance seen in both syndromes [125,126]. The recommended dietary allowance (RDA) for vitamin C varies by age and sex: 1–3 years: 15 mg/day; 4–8 years: 25 mg/day; 9–13 years: 45 mg/day; 14–18 years: 75 mg/day (males), 65 mg/day (females) [127,128]. Studies in other populations with similar oxidative stress profiles suggest that vitamin C supplementation improves vascular health and reduces oxidative biomarkers [129].

As a lipid-soluble antioxidant, vitamin E protects cell membranes from lipid peroxidation [130]. It is particularly relevant for reducing atherosclerosis risks in TS, where lipid metabolism is often disrupted [131], and improving insulin sensitivity in KS [100]. Combining vitamins C and E could enhance their benefits [132], offering a synergistic approach to managing oxidative stress-related cardiovascular and metabolic complications in both aneuploidies. The RDA for vitamin E is as follows: 1–3 years: 6 mg/day; 4–8 years: 7 mg/day; 9–13 years: 11 mg/day; 14–18 years: 15 mg/day (both sexes) [128]. In addition, increased intake of antioxidants, including vitamins C and E, beyond the usual dietary and supplemental levels has been linked to higher sperm count and improved motility in a sample of healthy non-smoking men, attenuating the impact of age on sperm motility, to some extent [133]. This evidence shows that antioxidant intake may be associated with semen quality in healthy males, a relevant consideration for KS.

Glutathione, a key intracellular antioxidant, plays a central role in maintaining redox balance [134]. Reduced glutathione levels have been implicated in oxidative damage [135]. Therefore, supplementation with N-acetylcysteine, a precursor of glutathione, may boost endogenous antioxidant defenses, helping to mitigate oxidative stress linked to neurocognitive deficits, vascular damage, and metabolic dysfunction [136,137]. While there are no specific DRVs for N-acetylcysteine, its pediatric use has been studied in conditions such as cystic fibrosis and respiratory disorders, suggesting a potential role in SCAs where oxidative stress is heightened.

Dietary polyphenols, such as flavonoids and catechins found in fruits, vegetables, and green tea, offer strong anti-inflammatory and antioxidant effects [138,139,140,141,142]. Their role in reducing adipose-related inflammation, improving endothelial function, and protecting against neurodegeneration makes them a promising dietary intervention for SCAs [143,144,145]. Encouraging polyphenol-rich diets in pediatric and adolescent patients may serve as a practical and non-invasive antioxidant strategy [146]. However, optimal intake levels remain undefined for children, and more research is needed to establish specific guidelines.

Carotenoids, a class of lipid-soluble antioxidants found in colorful fruits and vegetables, provide significant protection against oxidative damage by scavenging free radicals and quenching singlet oxygen [147]. Among them, beta-carotene, lutein, and zeaxanthin are particularly well-studied for their roles in maintaining cellular health and preventing oxidative damage to lipids, proteins, and DNA [148]. Beta-carotene serves as a precursor to vitamin A, contributing to immune function and vision health, while also reducing oxidative stress markers [149]. Lutein and zeaxanthin are crucial for protecting neural tissues, particularly the retina, against oxidative injury [150]. These carotenoids may offer added benefits for individuals with SCAs by targeting oxidative stress-related complications, such as neurodevelopmental deficits or cardiovascular risks [151]. The RDA for vitamin A (beta-carotene equivalent) in pediatrics is as follows: 1–3 years: 300 µg/day; 4–8 years: 400 µg/day; 9–13 years: 600 µg/day; 14–18 years: 900 µg/day (males), 700 µg/day (females).

Melatonin, beyond its role in regulating circadian rhythms, acts as a powerful antioxidant that scavenges ROS and enhances endogenous antioxidant enzyme activity [152]. Its neuroprotective effects could be particularly beneficial for addressing cognitive and psychological challenges in SCAs in pediatric populations [153,154]. For instance, melatonin supplementation may help mitigate oxidative damage in neural tissues [155,156] implicated in the neurodevelopmental and cognitive difficulties in SCAs. Moreover, studies have indicated distinct alterations in melatonin secretion in both KS, characterized by potential sleep disorders with elevated baseline melatonin levels that decrease with testosterone therapy, and TS, which shows abnormal day–night rhythms and desynchronized melatonin patterns that are unaffected by estrogen treatment [157,158,159,160,161,162]. While melatonin use in pediatrics remains an area of ongoing research, its safety profile in children has been well-documented in sleep disorders, suggesting potential applicability for SCA-related neurodevelopmental issues.

In general, encouraging balanced diets rich in natural antioxidants, including vitamins, minerals, and polyphenols, can provide a sustainable and holistic approach to reducing oxidative stress [163]. A Mediterranean-style diet, emphasizing fruits, vegetables, whole grains, and healthy fats, may be particularly effective in pediatric populations with SCAs [164]. Additionally, pairing antioxidants with syndrome-specific therapies can enhance outcomes. For instance, combining antioxidant supplementation with growth hormone and ERT in TS or TRT in KS may address both oxidative stress and the hormonal imbalances characteristic of each syndrome [165,166]. Early intervention may therefore mitigate neurocognitive issues, as well as reduce the risk of long-term cardiovascular and metabolic complications [167,168]. Among the various antioxidant strategies explored, we propose the following prioritization—summarized in Table 5—based on current evidence of efficacy, safety in pediatric populations, and relevance to SCA pathophysiology.

## 7. Conclusions

Oxidative stress emerges as a central, yet modifiable, contributor to the progression of SCAs in pediatric populations. This review underscores the importance of early antioxidant strategies, particularly in conjunction with syndrome-specific hormonal therapies, to mitigate long-term neurocognitive, metabolic, and cardiovascular complications. Despite promising evidence, several research gaps remain. Priority areas include longitudinal studies on antioxidant use in pediatric SCA cohorts; exploration of gene–antioxidant interactions, particularly involving *XIAP*, *SLC25A5*, and *MAO-A*; standardization of pediatric antioxidant dosing protocols; and trials assessing combined antioxidant and hormonal therapy in adolescents. A precision medicine approach integrating genetic, metabolic, and clinical factors is warranted to optimize care for children with SCAs.

## Figures and Tables

**Table 3:** Oxidative stress-related genes differentially expressed in SCAs: function, redox involvement, and pathological relevance. Abbreviations: ADHD, attention deficit hyperactivity disorder; ADP, adenosine diphosphate; ATP, adenosine triphosphate; BMP, bone morphogenetic protein; DUOX, dual oxidase; H_2_O_2_, hydrogen peroxide; KS, Klinefelter syndrome; MAPK, mitogen-activated protein kinase; NADPH, nicotinamide adenine dinucleotide phosphate; ROS, reactive oxygen species; SOD-2, superoxide dismutase-2; TF, transcription factor; TS, Turner syndrome.

Gene (Chromosomal Location)	Function	Biochemical Reaction	Damage Caused	Related Conditions
*XIAP* (Xq25)	Inhibits apoptosis; promotes SOD-2	Prevents caspase cascade, promotes mitochondrial integrity	Mitochondrial dysfunction, oxidative neuronal injury	Neurodevelopmental delay, cardiovascular issues (TS)
*SLC25A5* (Xq24)	Mitochondrial ADP/ATP exchange	Regulates mitochondrial respiration and ROS generation	ROS accumulation, metabolic inefficiency	Metabolic syndrome, energy dysregulation
*EGR1* (Xq13.1)	Transcription factor, oxidative stress response	Regulates apoptotic and redox-responsive genes	Abnormal proliferation and stress signaling	Growth abnormalities, altered cell cycle (TS)
*KLF4* (Xq24)	Anti-inflammatory and antioxidant TF	Regulates endothelial and immune antioxidant genes	Vascular inflammation, endothelial dysfunction	Atherosclerosis, vascular aging
*SOX11* (Xp22.3)	Osteogenic and neurodevelopmental TF	Modulates BMP signaling, regulates differentiation	Skeletal malformations, neurocognitive dysfunction	Turner-related skeletal dysplasia
*MAO-A* (Xp11.3; SRY-regulated)	Degrades monoamines, regulated by *SRY* (Yp11.2)	Produces H_2_O_2_ during catecholamine metabolism	Oxidative stress in neurons, H_2_O_2_ accumulation	ADHD, autism spectrum disorders
*GPX4* (19p13.11)	Detoxifies lipid peroxides	Reduces phospholipid hydroperoxides (prevents ferroptosis)	Ferroptotic cell death, especially in testis and neurons	Infertility (KS), cognitive decline
*NOX4* (11q14.3)	NADPH oxidase, generates ROS	Produces H_2_O_2_ from oxygen and NADPH	DNA/protein oxidative damage, tissue fibrosis	Thyroid disorders, diabetes, fibrosis
*GADD45B* (19p13.3)	Stress-responsive TF, MAPK modulator	Interacts with MAPKs, regulates apoptosis and DNA repair	Inflammation, susceptibility to neoplastic transformation	Inflammatory disease, colorectal cancer
*DUOXA1* (15q21.1)	DUOX maturation factor	Enables H_2_O_2_ production in thyroid hormone synthesis	ROS imbalance in thyroid; hypothyroidism	Congenital hypothyroidism

**Table 4:** Suggested antioxidant approaches by SCA type and age group. Abbreviations: HGAs, high-grade sex chromosome aneuploidies.

SCA Type	Age Group	Suggested Antioxidants	Adjunct Therapy	Clinical Focus
Turner Syndrome	Prepubertal	Vitamin C, vitamin E, polyphenol-rich diet	Cardiovascular monitoring	Endothelial health, metabolic prevention
Turner Syndrome	Adolescent	NAC, vitamin E	Estrogen replacement therapy	Oxidative stress reduction, lipid control
Klinefelter Syndrome	Prepubertal	Polyphenol-rich diet, multivitamins	Lifestyle advice	Prevent early metabolic impairment
Klinefelter Syndrome	Adolescent	NAC, CoQ10, vitamin E	Testosterone replacement therapy	Redox balance, neuroprotection
47,XXX/47,XYY/HGAs	All ages	General antioxidant-rich diet, vitamin C	Multidisciplinary surveillance	Neurocognitive support, systemic defense

**Table 5:** Prioritization of antioxidant interventions in pediatric SCAs. Abbreviations: ROS, reactive oxygen species.

Rank	Antioxidant	Main Mechanism of Action	Targeted Complications	Notes
1	Vitamin C + Vitamin E	ROS scavenging, lipid peroxidation prevention, vascular protection	Cardiovascular, metabolic	Synergistic effect, safe and well-studied
2	N-acetylcysteine (NAC)	Glutathione precursor, mitochondrial support	Neurocognitive, metabolic	Evidence from cystic fibrosis and others
3	Melatonin	ROS scavenger, upregulates antioxidant enzymes, neuroprotection	Neurodevelopmental	Also useful for sleep disturbances
4	Dietary polyphenols	Anti-inflammatory improves endothelial function	Metabolic, cardiovascular	Requires dietary adherence
5	Carotenoids	Free radical quenching, protects DNA, lipids, proteins	Neurodevelopmental, vision, immunity	Lutein and beta-carotene most relevant
